# Goitre multi-nodulaire révélant une tuberculose thyroïdienne

**DOI:** 10.11604/pamj.2015.22.220.8118

**Published:** 2015-11-10

**Authors:** Rim Lahiani, Madiha Mahfoudhi

**Affiliations:** 1 Service ORL, Hôpital Charles Nicolle, Tunis, Tunisie; 2 Service de Médecine Interne A, Hôpital Charles Nicolle, Tunis, Tunisie

**Keywords:** Goitre multi-nodulaire, tuberculose, granulome, multinodular goiter, tuberculosis, granuloma

## Image en medicine

La tuberculose de la thyroïde, le plus souvent de découverte fortuite, est rare même dans les zones d'endémie. Elle est exceptionnellement révélée par un goitre multi-nodulaire. La cervicotomie diagnostique n'a de place que devant les limites de la cytoponction. Selon l'orientation clinique, la recherche d'autres localisations tuberculeuses s'impose. Le traitement médical doit être complet et prolongé afin d’éviter toute rechute souvent plus difficile à prendre en charge. Patiente âgée de 49 ans, sans antécédents, hospitalisée pour exploration d'une volumineuse tuméfaction basi-cervicale antérieure évoluant depuis 8 mois, ayant augmenté progressivement de volume et une fièvre prolongée évoluant depuis un mois sans sueurs ni altération de l’état général. L'examen physique a objectivé une thyroïde augmentée de taille avec deux nodules thyroïdiens: lobaire droit de 3 cm et gauche de 7 cm. Les aires ganglionnaires étaient libres. La naso-fibroscopie a montré des cordes vocales mobiles. L’échographie cervicale a trouvé un goitre multi-nodulaire aux dépens des deux lobes. La radiographie du thorax et le bilan hormonal thyroïdien étaient normaux. Elle a bénéficié d'une thyroïdectomie totale. Certains diagnostics étaient évoqués dont un cancer ou un lymphome. L'examen anatomopathologique a objectivé des granulomes épithélioïdes et giganto-cellulaires avec présence de foyers de nécrose caséeuse diffus dans le parenchyme thyroïdien, alors que les nodules étaient de nature bénigne. Le diagnostic de tuberculose thyroïdienne a été posé. Elle n'avait pas d'autres localisations tuberculeuses. Elle a reçu un traitement antituberculeux pour une durée totale de 12 mois associé à une hormonothérapie substitutive à vie. L’évolution était favorable.

**Figure 1:**
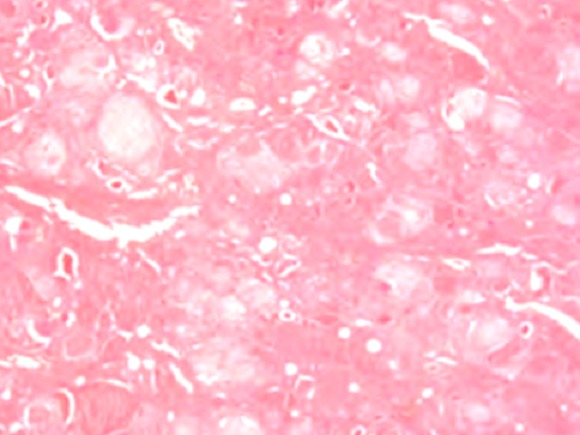
Granulomes épithéloïdes et giganto-cellulaires associés à des foyers de nécrose caséeuse diffus dans le parenchyme thyroïdien

